# Effect of Chitosan Nanoparticles Incorporating Antioxidants from *Salvia hispanica* L. on the Amaranth Flour Films

**DOI:** 10.17113/ftb.60.01.22.7144

**Published:** 2022-03

**Authors:** Gema Morales-Olán, María Antonieta Ríos-Corripio, Aleida Selene Hernández-Cázares, Placido Zaca-Morán, Silvia Luna-Suárez, Marlon Rojas-López

**Affiliations:** 1 Instituto Politécnico Nacional, Centro de Investigación en Biotecnología Aplicada, Ex Hacienda de San Juan Molino, Carretera estatal Santa Ines Tecuexcomac-Tepetitla km. 1.5, Tepetitla, 90700 Tlaxcala, Mexico; 2 CONACYT - Colegio de Postgraduados Campus Córdoba, Carretera Federal Córdoba-Veracruz km. 348, Amatlán de los Reyes, 94946 Veracruz, Mexico; 3 Colegio de Postgraduados Campus Córdoba, Carretera Federal Córdoba-Veracruz km. 348, Amatlán de los Reyes, 94946 Veracruz, Mexico; 4,Benemérita Universidad Autónoma de Puebla, Instituto de Ciencias, Ecocampus Valsequillo, 72960 Puebla, Mexico

**Keywords:** active films, biodegradable films, *Amaranthus hypochondriacus*, *Salvia hispanica* L., chitosan particles, response surface methodology

## Abstract

**Research background:**

Amaranth (*Amaranthus hypochondriacus*) flour produces films with excellent barrier properties against water vapor, allowing food preservation, but the mechanical properties are poor compared to synthetic films. One strategy to improve these properties is the incorporation of nanoparticles. The particles can also serve as a vehicle for the addition of antioxidant agents into the films. The objective of this work is to optimize the formulation for the preparation of amaranth flour films treated with antioxidant chia (*Salvia hispanica* L.) extract-loaded chitosan particles using response surface methodology (RSM).

**Experimental approach:**

Chitosan nanoparticles with the extract were synthesized by ionic gelation, and the films were made by the casting method. Three independent variables were assigned: amaranth flour (4-6%), glycerol (25-35%) and chitosan nanoparticles loaded with the chia extract (0-0.75%). We then evaluated the physical (thickness), mechanical (tensile strength, Young´s modulus and elongation), barrier (water vapor permeability, moisture and water solubility) and antioxidant properties of the films. The experimental results of the properties were analyzed using a Box-Behnken experimental design generating 15 runs with three replicates at the central point.

**Results and conclusions:**

Second and third order polynomial models were obtained from the ANOVA analysis of the evaluated responses, and high coefficients of determination were found (0.91–1.0). The water vapor permeability of the films was 0.82−2.39·10^-7^ (g·mm)/(Pa·s·m^2^), tensile strength was 0.33−1.63 MPa and antioxidant activity 2.24−5.65%. The variables had different effects on the films: glycerol negatively affected their properties, and the permeability values increased with increased amaranth flour content. The nanoparticles improved the mechanical, barrier and antioxidant properties of the films compared to the films without nanosystems. The optimal formulation was 4% amaranth flour, 25% glycerol and 0.36% chitosan nanoparticles. The optimized films had better mechanical (1.62 MPa) properties, a low water vapor permeability value (0.91·10^-7^ (g·mm)/(Pa·s·m^2^)) and moderate antioxidant activity (6.43%).

**Novelty and scientific contribution:**

The results show the effect of chitosan nanoparticles on the properties of amaranth flour films for the first time. The resulting equations are useful in the design of food packaging.

## INTRODUCTION

Films made with biopolymers are an ecological alternative for the preservation of food and pharmaceutical products. Proteins, carbohydrates and lipids are used in packaging, and plants are one of the main sources of these polymers (*1*). Amaranth (*Amaranthus hypochondriacus*) seeds are valued for their nutritional quality and functional properties. They contain carbohydrates (50%), proteins (16%) and lipids (8%) (*2*). Tapia-Blácido *et al*. (*3*) reported that amaranth flour forms films with good barrier properties against water vapor (2.58·10^-9^ (g·mm)/(Pa·s·m^2^)) and that the optimized film-forming solution must contain 20% glycerol (*4*) and the drying conditions must be at 50 ºC with 76.2% relative humidity (RH) (*5*). Chandla *et al.* (*6*) isolated starch from amaranth seeds obtaining translucent films with water vapor permeability (WVP) of 2.03−2.94·10^-10^ (g·mm)/(Pa·s·m^2^) and tensile strength of 2.3−2.6 MPa. Amaranth proteins have also been reported to have good filmogenic properties (*7*). However, although amaranth flour films have good barrier properties, their mechanical properties are poor compared to synthetic packaging. One current strategy to improve these properties is the incorporation of nanoparticles. The particles can form stronger interactions with the components of the polymer matrix and fill the empty pores in the films, thus achieving nanoreinforcement effects (*8*-*10*). In the literature, the addition of zinc oxide particles (*11*), nanoclay (*12*), silver and gold particles, cellulose (*13*) and chitosan particles (*14*) has proven helpful in reinforcing the films. Chitosan is one of the most widely used polymers in the production of nano- and microparticles. It is biocompatible, biodegradable, non-toxic and has antimicrobial properties (*15*, *16*). The addition of chitosan nanoparticles in films of tara gum, potato starch and fish gelatin improved the mechanical properties of the films (*9*, *10*, *17*). Villamán Diéguez *et al*. (*18*) reported the addition of nanoclay in amaranth flour films, but the nanoparticles did not improve their mechanical properties. Condés *et al*. (*19*) added corn starch nanocrystals to amaranth protein films and noted an increase in their tensile strength. However, there is no research about the addition of chitosan nanoparticles to amaranth flour films. Therefore, for the first time, this work studies the impact of the addition of chitosan nanoparticles on the properties of these films. The use of antioxidants from natural sources such as plant extracts has been increasing due to the damaging effects of synthetic antioxidants. Chia (*Salvia hispanica* L.) seed extract contains phenolic compounds such as kaempferol, quercetin, myricetin and chlorogenic acid at mass fractions of 0.509, 0.268, 0.018 and 0.045 mg/g of seed respectively (*20*). These compounds have good antioxidant properties and can be incorporated into films. The direct addition of antioxidants to the films affects their barrier and mechanical properties because phenolic compounds are hydrophilic and act as plasticizers (*21*). Nanoencapsulation can also be used as a vehicle for the incorporation of antimicrobial and antioxidant substances in the films to preserve food for a longer time using different mechanisms (*22*). The objective of this work is to evaluate the influence of various factors: the content of amaranth flour (4−6%), glycerol (25−35%) and chitosan particles loaded with antioxidant chia extract (0−0.75%) on the physical, barrier, mechanical and antioxidant properties of amaranth flour films treated with chitosan nanoparticles loaded with chia extract. The resulting optimal formulation produces films with the best characteristics.

## MATERIALS AND METHODS

### Materials

Chitosan (medium molecular mass, 75−85% deacetylated, apparent viscosity 0.2−0.8 Pa·s), sodium tripolyphosphate (TPP) with 85.0% purity grade, glacial acetic acid ≥99.7%, 2,2-diphenyl-1-picrylhydrazyl (DPPH), glycerol with ≥99.50% purity grade and ethanol ≥99.5% were acquired from Sigma-Aldrich, Merck (St. Louis, MO, USA).

### Amaranth and chia flour preparation

Amaranth and chia seeds were purchased from a local market in Puebla City, Mexico. Leaves and stones were manually removed from the seeds. The flour from both seeds was made in a food processor (Krups GX410011; Groupe SEB, Solingen, Germany), and a homogeneous particle size was obtained with a 60 mesh sieve. It was held at 4 °C until the films and the extract were prepared.

### Obtaining the antioxidant extract of chia seeds

Chia seed extract was obtained as reported by Morales-Olán *et al.* (*23*). Briefly, chia flour (0.5 g) was added to 80% aqueous ethanol solution (3 mL). The solution was stirred for 24 h at 25 °C and centrifuged (Multifuge™ X3; Thermo Scientific™, Madrid, Spain) at 1006.2×*g* for 10 min. The alcohol was then removed with a rotavapor (model R-114; Büchi Labortechnik AG, Flawil, Switzerland), and the water was removed by lyophilization (FreeZone 2.5-liter benchtop freeze dryer; Labconco, Kansas City, MO, USA).

### Preparation of chitosan nanoparticles loaded with chia extract

Chitosan nanoparticles loaded with the antioxidant extract of chia seeds were synthesized using the method reported by Morales-Olán *et al.* (*24*). Lyophilized chia extract (0.2 mg/mL) was mixed with the TPP solution (0.07% *m*/*V*). The TPP and chia extract solution was added dropwise in the chitosan solution (0.05% *m/V*) with stirring at room temperature. Chitosan particles were collected by centrifugation (Multifuge™ X3; Thermo Scientific™) at 4723.55×*g* for 30 min at 25 °C. Samples were then washed three times with deionized water, lyophilized and stored at 4 °C. The empty particles without the extract were made with the same method but without the addition of the chia extract to the TPP solution.

#### Encapsulation efficiency and loading capacity of the particles

The encapsulation efficiency was determined according to the method proposed by Morales-Olán *et al*. (*24*). The supernatant of the particles was analyzed by UV-Visible BioTek Epoch^TM^ 2 microplate spectrophotometer (Agilent Technologies, Winooski, VT, USA) at 320 nm. At this wavelength, the chia extract showed maximum absorption and the precursors (chitosan and TPP) had no absorbance. The concentration of the chia extract in the supernatant was calculated with a calibration curve (0.0−1.5 mg/mL, R=0.99) using the following equation:



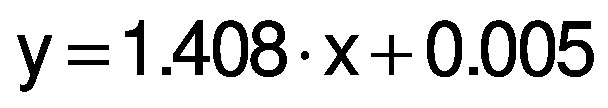



 The encapsulation efficiency and loading capacity of the particles were calculated using the following equations respectively:



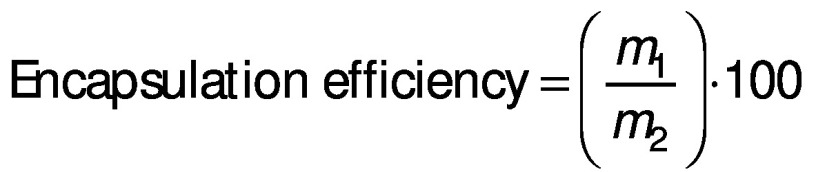



and



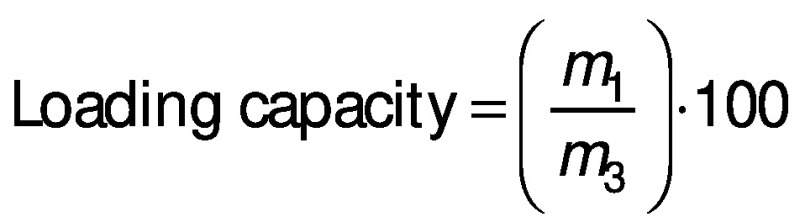



where *m*_1_ is the mass of the loaded chia extract, *m*_2_ is the mass of initial chia extract, and *m*_3_ is the mass of sample.

### Particle morphology and size

The morphology and size of the particles with and without extract were evaluated using field emission scanning electron microsope (JSM-7610F; JEOL, Tokyo, Japan) and an electron backscatter diffraction (EBSD) detector (voltage acceleration: 2.0 KV; Oxford Instruments plc, Abingdon, UK) and secondary electron detector (SEI). The particles were conditioned on carbon tape and coated with Au/Pd. The images were analyzed with the ImageJ program v. 1.52r to determine the average size of the particles (*25*).

### Morphology of the films

The morphology of the amaranth flour film surface was examined using a scanning electron microscope (Vega TS-5136SB; Tescan, Kohoutovice, Czech Republic) operating in low vacuum mode. Film samples were mounted on an aluminum base with carbon tape.

### Structural characterization of the particles and the films by FTIR

The precursors of the synthesis of the particles (chitosan and TPP), chia extract, empty particles, particles loaded with chia extract, and the films (with and without nanoparticles) were characterized with Fourier transform infrared (FTIR) spectroscopy (Vertex 70v; Bruker, Bremen, Germany) with an attenuated total reflectance (ATR) accessory. Measurements were made from 4000 to 500 cm^-1^.

### Preparation of edible films with chitosan nanoparticles

The films were produced by a casting method. Suspensions of the amaranth flour (4, 5 and 6%, *m/V*) in water were homogenized for 25 min, and the pH was adjusted to 10 using 2 M NaOH. The filmogenic solutions were then heated at 80 °C for 15 min and different mass fractions of glycerol (25, 30 and 35%, dry mass basis) were added. The amaranth flour and glycerol solutions were then centrifuged (Multifuge™ X3; Thermo Scientific™) at 698.75×*g* for 10 min. The chitosan nanoparticles loaded with the chia extract (0, 0.375 and 0.75%, based on the mass of amaranth flour) were dispersed in 500 µL of water and vortexed for 30 min. Samples were then added to the filmogenic solution. The dispersions were stirred for 10 min at room temperature. The filmogenic solutions were placed in Petri dishes with silicone molds (12.57 cm^2^) and dried at 40 °C for 12 h. The nanocomposite films were conditioned at 58% relative humidity (RH) using saturated NaBr solutions for 48 h before characterization.

### Film characterization

#### Thickness

Film thickness was measured with a digital micrometer (MDC-1 MX; Mitutoyo, Kawasaki, Japan). Ten measurements were made on each film at different points. Average values ​​were used to calculate the mechanical and barrier properties.

#### Moisture content, water solubility and water vapor permeability

For the determination of moisture content, the film samples were cut (2.0 cm×2.0 cm) after conditioning and placed in porcelain capsules weighed before and after oven drying. The moisture content was determined after drying in an oven at 105 °C for 24 h, and the percentage of mass loss was calculated based on the initial mass. The water solubility was measured by immersion of the film (2.0 cm×2.0 cm) in distilled water at 25 °C for 24 h (*17*). The solution was filtered through a previously weighed filter paper and placed in the oven at 105 °C for 24 h. The water solubility was obtained by difference in the initial and final mass of the film.

The water vapor permeability (WVP) was determined using the cup method according to ASTM E-96/E96M-16 standard (*26*) with the slightly modified method of Beristain-Bauza *et al*. (*27*). Beakers with a diameter of 25 mm were used. Anhydrous silica (0% RH) was added to the beakers, the films were secured with Parafilm® paper and then placed inside a desiccator with saturated NaCl solution (75% RH) at 25 °C. The mass of water transferred through the film was determined from the mass gain every 24 h for 120 h. Three determinations were made for each film. The WVP ((g·mm)/(Pa·s·m^2^)) was calculated using the following equation:



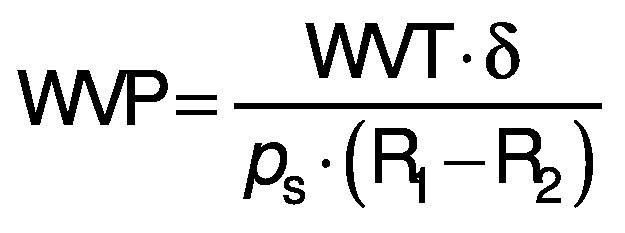



where the water vapor transmission (WVT) (g/(Pa·s·m^2^)) was calculated with the slope of the linear regression of the mass gain *versus* time divided by the cup area (*A*/m^2^), *δ* is film thickness in mm, *p*_s_ is saturation vapor pressure at 25 °C in Pa, R_1_ is the RH of saturated NaCl solution expressed as a fraction, and R_2_ is the RH of desiccant. All three properties were measured in triplicate.

#### Tensile strength, elongation and Young´s modulus

A texture analyzer (EZ test; Shimadzu, Kyoto, Japan) was used to measure the mechanical properties according to ASTM D882-18 (*28*). The films were cut into strips (2.0 cm×5.0 cm) after conditioning. The initial grip spacing used was 45 mm, and the crosshead speed 0.5 mm/s. The tensile strength was determined from stress-strain curves.

The elongation was calculated using the following equation:



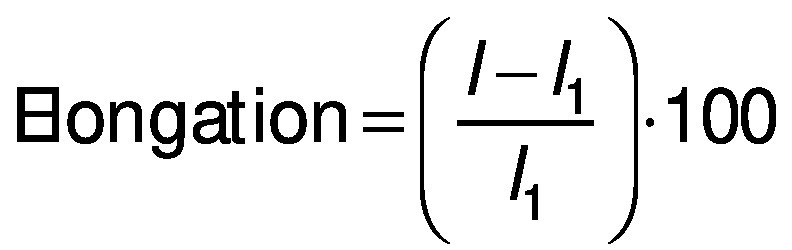



where *l*_1_ is the initial length of the film, and *l* is the length of the film at breaking point.

Young’s modulus was calculated by considering the steepest slope of the initial linear portion of this curve.

#### DPPH radical scavenging activity

The antioxidant capacity of the films was measured as DPPH radical scavenging activity. The films (25 mg) were mixed with 100 mM DPPH methanol solution for 30 min in the dark with agitation. The sample was then centrifuged (Multifuge™ X3; Thermo Scientific™) at 1006.2×*g* for 1 min. The absorbance of supernatant was measured at 517 nm. The antioxidant activity was determined in triplicate using the following equation:



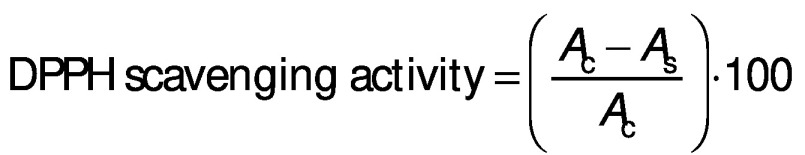



where *A*_c_ is the absorbance of the control, and *A*_s_ is the absorbance of the samples (films).

### Experimental design and statistical analysis

The experimental results of the measurement of the film properties were analyzed by a Box-Behnken experimental design. Three independent variables with three levels were used, thus generating 15 runs with three replicates at the central point. The independent variables were the amaranth flour content (x_1_), glycerol content (x_2_) and chitosan nanoparticles loaded with chia extract (x_3_). The levels of the independent variables were selected based on preliminary experiments. The coded levels for these variables were amaranth flour content: 4  (-1), 5 (0) and 6% (+1), glycerol content: 25 (-1), 30 (0) and 35% (+1), and content of chitosan particles with chia seed extract: 0 (-1), 0.375 (0) and 0.75% (+1).

The response variables were the physical (thickness), barrier (moisture content, water solubility and WVP), mechanical (tensile strength, elongation and Young’s modulus), and antioxidant (DPPH radical scavenging activity) properties of the films. Response surface methodology (RSM) was used to obtain the surface plots associated with the properties of the films to evaluate the influence of the independent variables on the properties. Multiple regression calculations were performed with Design Expert® software v. 13 (*29*). All processes were verified: orders of mean, the linear polynomial, linear polynomial with two-factor interaction, quadratic and cubic model. The quadratic and cubic models were selected because of the insignificant lack of fit and a high R^2^. The cubic model was reduced based on low p-value and high mean square values to generate a simplified model. The effect of each independent variable on the properties was determined, and the experimental data were fitted to the following quadratic and cubic models for each response (Y), respectively:



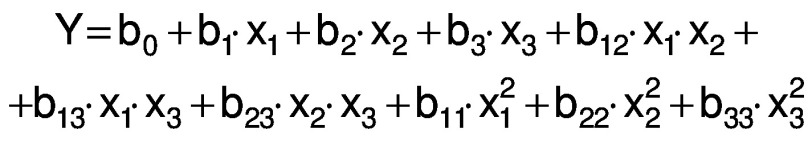





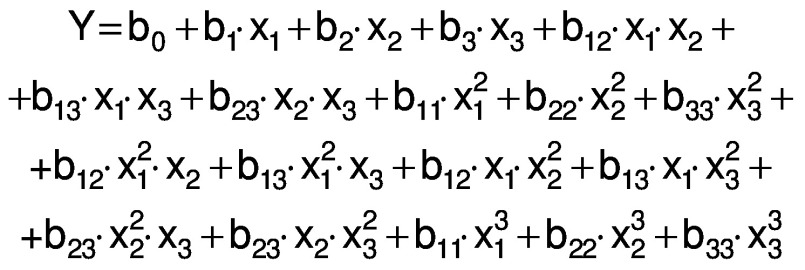



where Y is the response, x_1_ is the amaranth flour content, x_2_ is glycerol content and x_3_ the content of chitosan nanoparticles loaded with chia extract. Term b_0_ is the intercept, b_1_, b_2_ and b_3_ are the linear terms, b_11_, b_22_ and b_33_ are the quadratic and cubic terms, and b_12_, b_13_ and b_23_ are the interaction regression coefficient terms. A response surface plot was made from the regression equations for each property. The significant differences in the experimental data were obtained with ANOVA.

## RESULTS AND DISCUSSION

### Characterization of the chitosan nanoparticles loaded with chia extract

**Fig. 1:**
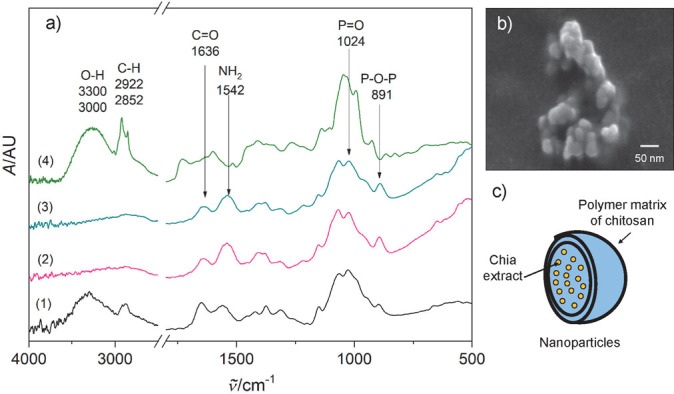
Characterization of chitosan nanoparticles loaded with chia extract: a) FTIR spectra of: chitosan (1), empty particles (2), loaded particles (3) and chia seed extract (4), b) SEM micrograph of the chitosan particles loaded with chia extract, and c) schematic representation of the structure of the loaded synthesized particles

Once the extract of *Salvia hispanica* L. was obtained, it was incorporated into the chitosan nanoparticles, which were then characterized. Fig. 1a shows the FTIR spectra of the chia extract, empty and loaded particles and the precursor (chitosan), followed by the scanning electron microscope image of the loaded extract in Fig. 1b, and schematic presentation of its synthesized particles in Fig. 1c.

Characteristic signals of the phenolic compounds were observed in the spectra for chia extract (Fig. 1a (4)). In the 3300−3000 cm^-1^ region, the broad band corresponds to the stretching vibrations of the O-H bonds. The next two bands show the stretching vibrations of the C-H bonds at 2922 and 2852 cm^-1^. Signals from the carbonyl and aromatic groups appear from 1600 to 1400  cm^-1^. These groups are considered an important part of the structure of phenolic compounds.

The CH spectrum (Fig. 1a (1)) is characterized by signals from 3700 to 2800 cm^-1^. These signals correspond to the stretching vibrations of the O-H, N-H and C-H bonds (*15*). The symmetric stretching vibrations of the carbonyl (C=O) and amino (NH_2_) groups of the chitosan molecule are seen at 1648 and 1557 cm^-1^ (*30*). These bands move to 1636 and 1542 cm^-1^ respectively in the spectrum of chitosan nanoparticles (Fig. 1a (2 and 3)). The band of the amino group increased, and the signals located at 1024 and 891 cm^-1^ indicated the stretching vibration of the phosphate group by the incorporation of TPP and the formation of the particles (*15*, *30*). No differences were observed between the spectra of the empty and loaded particles (Fig. 1a (2 and 3)). These results suggest that the added extract is within the particle; thus, nanocapsules were synthesized with this method (Fig. 1c).

The particles loaded with the chia extract have a spherical morphology and an average size of (39.7±8.4) nm (Fig. 1b). The encapsulation efficiency of the chia extract in the chitosan particles was (93.0±4.5) %. This value was higher than that reported in the encapsulation of chlorogenic and ferulic acids in chitosan particles (*31*, *32*). The loading capacity of the particles was (16.2±1.5) %. Chia extract was retained more in the chitosan particles than resveratrol and eugenol (*33*, *34*). These particles were added to the amaranth flour films.

### Structural analysis, morphology and appearance of the films

Films without particles and nanocomposites were also analyzed to observe the interactions between amaranth flour and nanoparticles.

**Fig. 2:**
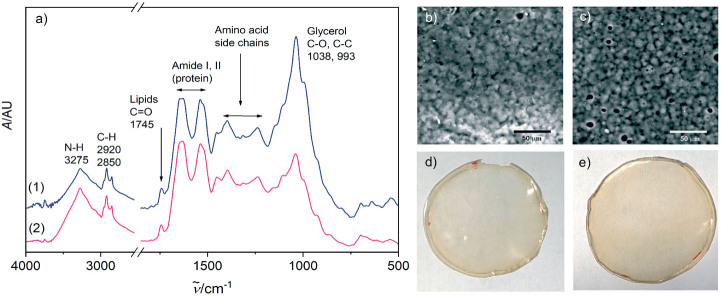
Amaranth flour films: a) FTIR spectra: 4% amaranth flour, 30% glycerol and 0% chitosan nanoparticles (1) and 4% amaranth flour, 25% glycerol and 0.375% chitosan nanoparticles (2), b and c) SEM images of (1) and (2) respectively, and d and e) photographs of the film with and without chitosan nanoparticles respectively

Fig. 2 shows the FTIR spectra of the film prepared with 4% amaranth flour, 30% glycerol and 0% chitosan nanoparticles (1), and the film prepared with 4% amaranth flour, 25% glycerol and 0.375% chitosan nanoparticles (2). The main vibrational features include a band at 3275 cm^-1^ related to the N-H vibration of proteins. Other bands at 2920 and 2850 cm^-1^ were from the C-H stretching vibrations of proteins, carbohydrates and glycerol. The next spectral features include a band at 1741 cm^-1^ that arises from the C=O stretching vibration of the carbonyl group of lipids in the amaranth flour. Other intense bands centered at 1637 and 1540 cm^-1^ are related to the amide I (C=O stretching) and amide II (N-H bending) functional groups of proteins, respectively (*35*). The band at 1452 cm^-1^ corresponds to methyl methylene groups from lipids and proteins; another band at 1396 cm^-1^ arises from the COO^-^ stretching of amino acid side chains. The band at 1238 cm^-1^ is related to the P=O stretching of PO_2_ phosphodiester groups from phosphorylated molecules. The next absorption bands at 1110, 1038, 993, 923 and 854 cm^-1^ are due to the C-O and C-C vibrations in the skeleton of the glycerol molecule. No significant differences were observed between the spectra of the amaranth flour films with and without nanoparticles. This may be due to the low concentration of the used nanoparticles. Other studies reported that the lack of significant changes in the FTIR spectra of films with and without chitosan nanoparticles is because the polymeric matrix and the nanoparticles interact only *via* hydrogen bonds (*36*).

Figs. 2b and 2c show SEM micrographs of the surface of the amaranth flour films without and with nanoparticles. The granular appearance of the polymeric matrix of both films is attributed mainly to proteins in the flour. Although the concentration of chitosan nanoparticles is low (0.375%), interactions between them and proteins could occur, thus increasing the granular morphology, contrary to films without chitosan nanoparticles. Some irregularities in the polymeric matrix such as pinholes formed during the drying of the filmogenic solution were also observed. The films were homogeneous, translucent and slightly yellow. Visually, no differences were seen between films with and without nanoparticles (Figs. 2d and 2e).

Statistical analysis of the effect of the independent variables on the properties of the amaranth flour films are described below.

### Statistical analysis and model fitting of Box-Behnken design

The Box-Behnken design results helped to analyze the effect of the independent variables (factors) including the amaranth flour content (4−6%), glycerol (25−35%), and chitosan particles loaded with the antioxidant extract of chia (0−0.75%). Outcome variables included the physical, barrier, mechanical and antioxidant properties of amaranth flour films (Table 1 and Table 2). There were 15 average measurements of the dependent variables used to obtain fitting parameters and ANOVA.

**Table 1:** Responses of the parameters used in the experimental design

Run	Independent variable (Factor)/Coded variable			Dependent variable (Response)							
(*m*(amaranth flour)/*V*(water))/%, x_1_	*w*(glycerol)/%, x_2_	*w*(chitosan particle)/%, x_3_	*δ*/mm	WVP·10^-7^/((g·mm)/(Pa·s·m^2^))	*w*(moisture)/%	Water solubility/%	Tensile strenght/MPa	Young´s modulus/MPa	Elongation/%	DPPH scavenging activity/%
1	4 (-1)	25 (-1)	0.375 (0)	(0.069±0.007)ª	(1.08±0.08)ª	(20.11±0.01)ª	(62.8±0.1)ª	(1.6±0.1)ª	(0.3±0.1)ª	(38.9±0.5)ª	(6.6±0.5)ª
2	6 (1)	25 (-1)	0.375 (0)	(0.094±0.005)^b^	(1.77±0.06)^b^	(22.7±0.5)^ab^	(58.2±0.2)^b^	(1.1±0.2)^b^	(0.16±0.05)^ab^	(34.4±0.5)ª	(3.2±0.8)^ab^
3	4 (-1)	35 (1)	0.375 (0)	(0.069±0.002)ª	(1.5±0.1)^ab^	(25.3±0.3)^bc^	(60.2±0.1)^ac^	(1.07±0.03)^b^	(0.11±0.00)^b^	(35.6±0.4)ª	(2.6±0.2)^bc^
4	6 (1)	35 (1)	0.375 (0)	(0.103±0.009)^b^	(2.4±0.2)^b^	(28.9±0.1)^d^	(53.4±0.3)^d^	(0.35±0.00)^c^	(0.05±0.04)^b^	(56.7±0.0)^b^	(6.4±0.5)^ab^
5	4 (-1)	30 (0)	0.000 (-1)	(0.070±0.007)ª	(0.9±0.3)ª	(28.9±0.2)^d^	(53.47±0.09)^d^	(0.44±0.00)^c^	(0.03±0.00)^b^	(75.6±0.6)^c^	(2.2±0.2)^bd^
6	6 (1)	30 (0)	0.000 (-1)	(0.111±0.004)^b^	(2.0±0.2)^b^	(18.3±0.5)ª	(63.35±0.09)ª	(0.55±0.00)^cd^	(0.03±0.00)^b^	(34.4±0.5)ª	(3.5±0.2)^ab^
7	4 (-1)	30 (0)	0.750 (1)	(0.070±0.003)ª	(1.3±0.3)^ab^	(25.5±0.4)^be^	(56.49±0.04)^bcd^	(0.68±0.06)^c^	(0.04±0.01)^b^	(46.7±1.0)^ab^	(6.1±1.0)^ab^
8	6 (1)	30 (0)	0.750 (1)	(0.095±0.009)^b^	(2.2±0.3)^b^	(26.2±0.3)^cdef^	(55.00±0.07)^bd^	(0.6±0.1)^ce^	(0.04±0.01)^b^	(41.1±0.5)ª	(6.2±1.0)^ab^
9	5 (0)	25 (-1)	0.000 (-1)	(0.101±0.004)^b^	(1.31±0.07)^ab^	(23.8±0.4)^bfg^	(61.84±0.09)^ab^	(0.89±0.04)^bdef^	(0.09±0.00)^b^	(44.4±0.5)ª	(5.1±0.7)^ab^
10	5 (0)	35 (1)	0.000 (-1)	(0.104±0.005)^b^	(2.38±0.07)^b^	(24.4±0.4)^bfh^	(58.8±0.7)^ab^	(0.43±0.08)^c^	(0.03±0.01)^b^	(35.6±0.5)ª	(8.6±1.0)^a^
11	5 (0)	25 (-1)	0.750 (1)	(0.072±0.003)ª	(0.8±0.1)ª	(22.14±0.00)^acgh^	(59.4±0.5)^ab^	(0.5±0.2)^cf^	(0.11±0.00)^ab^	(45.6±0.7)ª	(5.7±0.1)^acd^
12	5 (0)	35 (1)	0.750 (1)	(0.074±0.009)ª	(1.27±0.04)^ab^	(30.3±0.4)^d^	(55.8±0.2)^bd^	(0.33±0.00)^cf^	(0.04±0.04)^b^	(61.1±0.8)^b^	(5.2±0.5)^acd^
13	5 (0)	30 (0)	0.375 (0)	(0.084±0.007)^ab^	(1.5±0.1)^ab^	(26.6±0.5)^cdegh^	(57.6±0.4)^ab^	(0.6±0.2)^cf^	(0.05±0.02)^b^	(35.1±0.0)^a^	(7.6±0.2)ª
14	5 (0)	30 (0)	0.375 (0)	(0.078±0.004)^ab^	(1.7±0.2)^ab^	(26.8±0.5)^cdegh^	(57.6±0.6)^bcd^	(0.65±0.03)^cf^	(0.03±0.02)^b^	(35.1±0.0)^a^	(7.6±0.4)ª
15	5 (0)	30 (0)	0.375 (0)	(0.084±0.002)^ab^	(1.6±0.4)ª	(25.1±0.4)^beg^	(57.6±0.7)^bcd^	(0.65±0.01)^cf^	(0.03±0.00)^b^	(35.1±0.5)ª	(7.6±1.0)ª

The results show the mean value±S.D. Results with the same letter in superscript in the same column are not significantly different (p<0.05) according to Tukey’s test. *δ*=thickness, WVP=water vapor permeability

**Table 2:** ANOVA obtained for the model ﬁtting

Response	Source	Sum of squares	df	Mean square	F-value	p-value	Model summary
Thickness	Quadratic model	0.00282	9	0.00031	5.84	0.033*	R^2^=0.91 Adj R^2^=0.75
	Residual	0.0003	5	0.0001	-	-	
	Lack of fit	0.0002	3	0.0001	6.92	0.129	
	Pure error	0.00002	2	0.000012	-	-	
	Total	0.0031	14	-	-	-	
Water vapour permeability	Reduced cubic model	3.39·10^-14^	8	4.24·10^-15^	16.48	0.0015*	R^2^=0.95 Adj R^2^=0.89
	Residual	1.54·10^-15^	6	2.54·10^-16^	-	-	
	Lack of fit	1.35·10^-15^	4	3.38·10^-16^	3.53	0.2329	
	Pure error	1.91·10^-16^	2	9.58·10^-17^	-	-	
	Total	3.5·10^-14^	14	-	-	-	
Moisture content	Reduced cubic model	143.39	8	17.92	13.72	0.0025*	R^2^=0.94 Adj R^2^=0.87
	Residual	7.84	6	1.31	-	-	
	Lack of fit	6.03	4	1.51	1.67	0.4077	
	Pure error	1.81	2	0.9033	-	-	
	Total	151.231	14	-	-	-	
Water solubility	Reduced cubic model	132.55	11	12.05	17112.45	<0.0001*	R^2^=1.0 Adj R^2^=0.99
	Residual	0.021	3	0.0007	-	-	
	Lack of fit	0.0021	1	0.0021	-	-	
	Pure error	0.0000	2	0.000	-	-	
	Total	132.55	14	-	-	-	
Tensile strenght	Reduced cubic model	1.65	12	0.1377	734.45	0.0014*	R^2^=0.99 Adj R^2^=0.99
	Residual	-	-	-	-	-	
	Lack of fit	-	-	-	-	-	
	Pure error	0.0004	2	0.0002	-	-	
	Total	1.65	14	-	-	-	
Young´s modulus	Quadratic model	0.0544	9	0.0060	5.01	0.0455*	R^2^=0.90 Adj R^2^=0.72
	Residual	0.0060	5	0.0012	-	-	
	Lack of fit	0.0059	3	0.0020	25.12	0.0385	
	Pure error	0.0004	2	0.0001	-	-	
	Total	0.0604	14	-	-	-	
Elongation	Reduced cubic model	2037.34	11	185.21	29.75	0.0087*	R^2^=0.99 Adj R^2^=0.95
	Residual	18.67	3	6.22	-	-	
	Lack of fit	18.67	1	18.67	-	-	
	Pure error	0.0000	2	0.0000	-	-	
	Total	2056.01	14	-	-	-	
DPPH radical scavenging activity	Reduced cubic model	54.55	11	4.96	119.22	0.0011*	R^2^=0.99 Adj R^2^=0.98
	Residual	0.1248	3	0.0416	-	-	
	Lack of fit	0.1248	1	0.1248	-	-	
	Pure error	0.0000	2	0.0000	-	-	
	Total	54.68	14	-	-	-	

*significant at 5% level

The ANOVA analysis showed that the quadratic regression models of the thickness and Young’s modulus responses were statistically significant (p<0.05). Responses from WVP, moisture content, water solubility, tensile strength, elongation and antioxidant activity were found statistically significant *via* reduced cubic regression model (p<0.05). The lack of fit for the F values of the thickness, WVP, moisture content and Young’s modulus responses suggests that the fit is not significant in relation to the pure error. There is a 12.9, 23.29, 40.77 and 3.85%, respectively, chance of a lack of adjustment of the F value due to noise. The insignificance of the lack of fit suggests that the model fits the experimental data.

All response variables had a high R^2^, *i.e.* there is a close correlation between the predicted and experimental values. Therefore, these models are suitable for predicting the responses for films made with amaranth flour added to chitosan nanoparticles loaded with the chia extract. The regression analysis for model fitting is presented in Table 3.

**Table 3:** Regression coefficients for response variables in the experimental design

Regression coefficient	Thickness	Water vapour permeability	Moisture content	Water solubility	Tensile strenght	Young´s modulus	Elongation	DPPH radical scavenging activity
Constant	0.121292	-1.22·10^-6^	-698.06134	97.38625	90.05802	4.15794	99.55865	50.29401
FTB-60-52-g1.eps	0.003183*	4.36·10^-7^*	139.01243*	1.07500*	-21.23230	-0.456819	104.34144*	12.58129*
FTB-60-52-g2.eps	-0.005931	3.96·10^-9^*	50.05832*	-4.23500*	-5.15362*	-0.189826*	18.12657*	-5.54685
FTB-60-52-g3.eps	0.012883*	3.77·10^-6^*	-65.32016*	169.5966*	13.54740*	0.351500	467.51044*	-167.3694*
FTB-60-52-g4.eps	0.000331	-4.18·10^-8^	-	0.730000*	0.983047*	0.038400	4.54490*	-2.28180*
FTB-60-52-g5.eps	0.000070	-	-0.836385	0.059100*	0.072493*	0.002862*	-0.418315	0.068517*
FTB-60-52-g6.eps	0.031193	-	-	-176.8711*	-1.97298*	-0.285422	632.92385*	-46.39294*
FTB-60-52-g7.eps	0.000433	1.09·10^-9^	-9.67310	0.270500*	1.09397*	0.001865	1.27780*	0.358277*
FTB-60-52-g8.eps	-0.010704	-1.44·10^-6^	7.59989*	-34.00667*	-5.25476*	-0.010033	108.16426*	-0.738758
FTB-60-52-g9.eps	-0.000267	-8.30·10^-9^	1.00902*	-0.088000*	0.034561*	-0.002340	-74.96145*	13.54881*
FTB-60-52-g10.eps	-	-	-	-0.038500*	-0.032136*	-	-	-
FTB-60-52-g11.eps	-	1.43·10^-7^*	-	-	0.514453*	-	-	-
FTB-60-52-g12.eps	-	-	0.162046*	-	-0.013027*	-	-	-
FTB-60-52-g13.eps	-	-	-	35.23556*	-	-	-112.6149*	-
FTB-60-52-g14.eps	-	-	-	-	-	-	1.30368*	-0.251606*
FTB-60-52-g15.eps	-	-	-	-	-	-	-	1.34128*

*significant at 5% level

The regression coefficients of WVP, moisture content, water solubility and elongation of the films were studied for the factors: amaranth flour, glycerol and chitosan nanoparticles loaded with chia extract, and they were found significant (p<0.05). In the thickness and antioxidant activity responses, the coefficients for amaranth flour and chitosan nanoparticles loaded with chia extract were significantly correlated. The tensile strength was significantly affected by the glycerol and nanoparticle content, while Young’s modulus was only affected by the glycerol content. The estimated models created for the response variables can be described using the following equations:



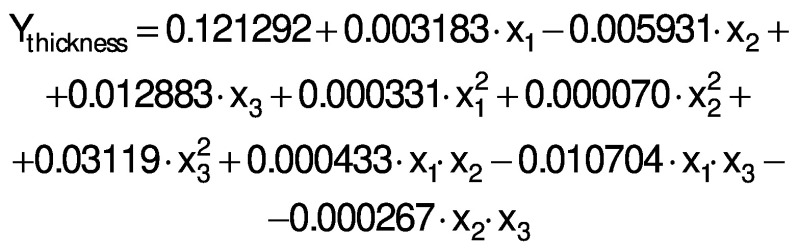





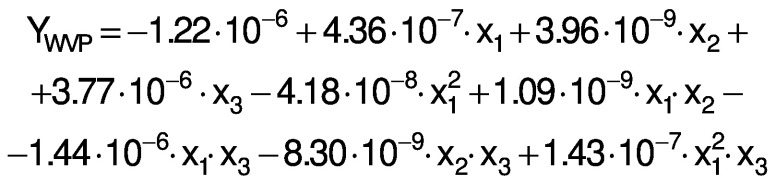





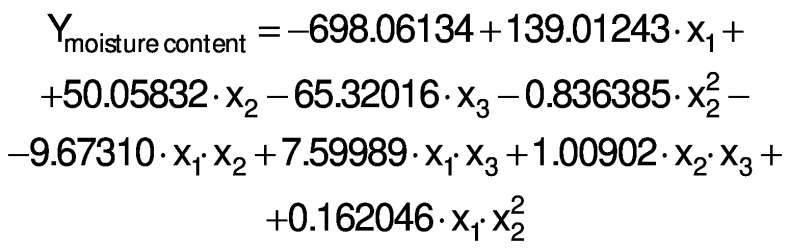





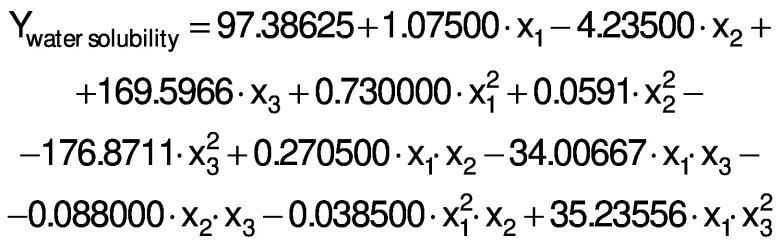





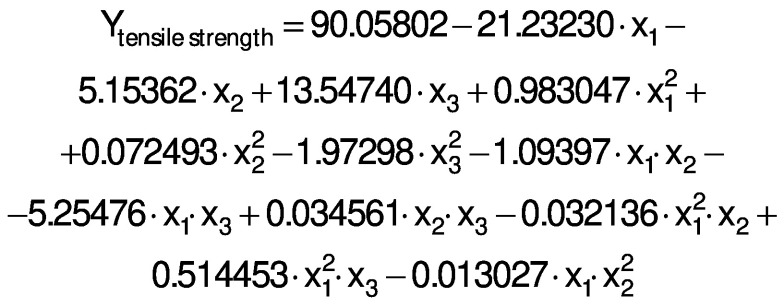





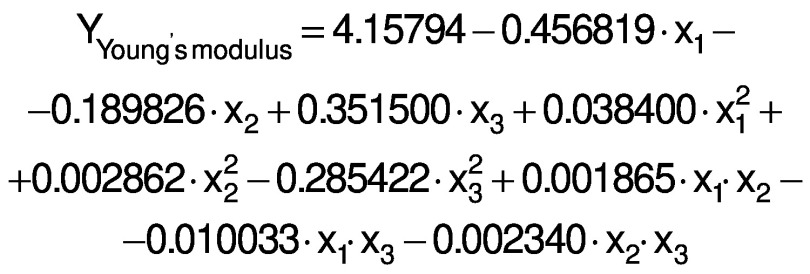





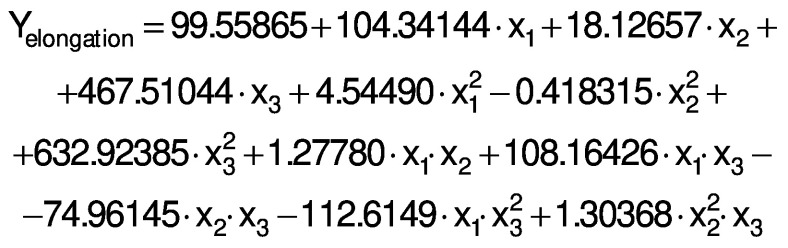





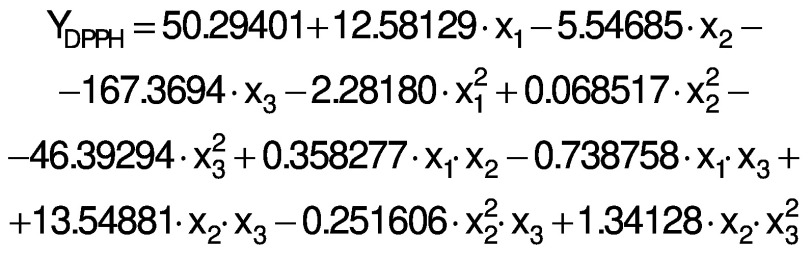



The response surface graphs between two independent variables were elaborated to investigate the relationship between the independent variables and the properties of the films (physical, barrier, mechanical and antioxidant). The behavior found in one of the evaluated properties is detailed below.

### Analysis of response surface

#### Physical properties

**Fig. 3:**
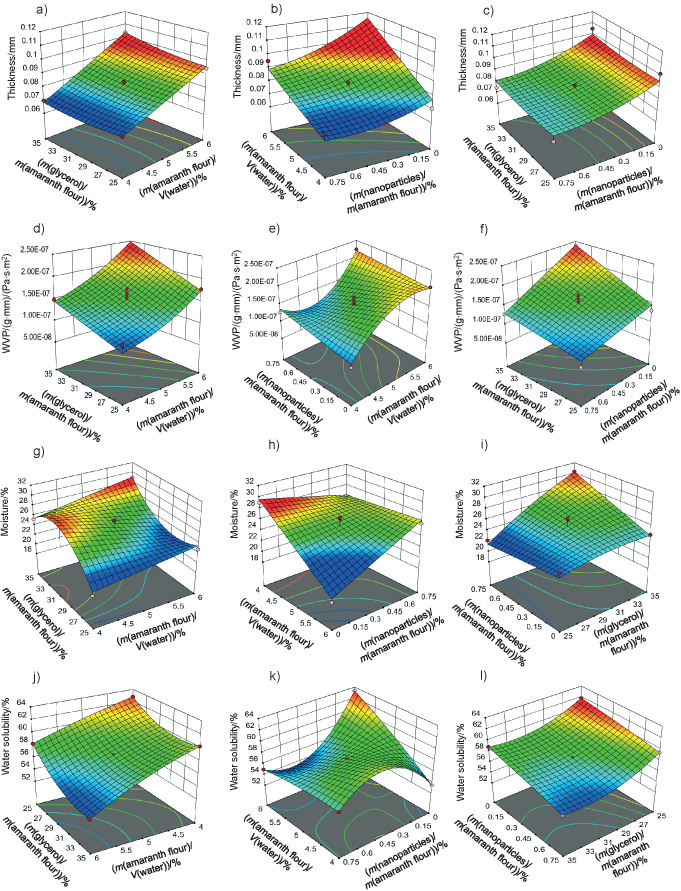
Response surface plots of the combined effects of independent variables on the: a-c) thickness, d-f) water vapor permeability, g-i) moisture content, and j-l) water solubility of amaranth flour films with chitosan particles loaded with chia extract

The thickness of the films varied from 0.06 to 0.11 mm (Table 1), similar to those reported by Tapia-Blácido *et al*. (*5*) for amaranth flour films without nanoparticles, and by Villamán Diéguez *et al*. (*18*) for amaranth flour films incorporating nanoclay. The values are lower than reported by Chandla *et al.* (*6*) for films made with amaranth starch. The amaranth flour and chitosan nanoparticles loaded with chia extract had a signiﬁcant impact on the thickness of the ﬁlm (Table 3). Fig. 3 shows the response surface plots of the combined effects of independent variables on the thickness (Figs. 3a-c), water vapor permeability (Figs. 3d-f), moisture content (Figs. 3g-i) and water solubility (Figs. 3j-l). The thickness increased with the increase of amaranth flour content because there are more dissolved solids in the same volume of the filmogenic solution (Fig. 3a).

The results agree with Singh *et al*. (*16*) and Thakur *et al*. (*37*) for films made with chitosan and pea starch. In contrast, the increase in the mass fraction of the nanoparticles led to a decrease in the film thickness (Fig. 3c). Shariatinia and Zahraee (*38*) explained that the particles may not be interacting with the polymeric chains of the filmogenic matrix. They only intercalated with each other, thus causing a decrease in thickness.

Antoniou *et al*. (*9*) found no significant differences in the thickness with increasing nanoparticle mass fraction in chitosan films. Hosseini *et al*. (*10*) described an increase in the thickness as the mass fraction of nanoparticles in fish gelatin films increased. Variations in the thickness of the films treated with nanoparticles are due to the applied manufacturing processes and the characteristics of the polymeric matrices.

#### Barrier properties

Films with lower WVP values suggest good food preservation capacity. The WVP of the amaranth flour films with chitosan nanoparticles loaded with chia extract were 0.82−2.39·10^-7^ (g·mm)/(Pa·s·m^2^) (Table 1). These values are better than those of the films made with tapioca flour, cellulose and sodium caseinate (*39*). However, they were higher than those reported by Tapia-Blácido *et al*. (*5*) and Chandla *et al*. (*6*) for flour and amaranth starch films without nanoparticles as well as by Villamán Diéguez *et al*. (*18*) for amaranth flour films incorporating nanoclay. The WVP of the elaborated films were also higher than those reported in synthetic films of polyethylene terephthalate (1.3·10^-9^ (g·mm)/(Pa·s·m^2^)) and polypropylene (4.7·10^-9^ (g·mm)/(Pa·s·m^2^) (*1*). The WVP was significantly affected by the content of amaranth flour, glycerol and nanoparticles (Table 3). The permeability values increased with the increased content of amaranth flour and glycerol (Fig. 3d). Similar results were reported by Thakur *et al*. (*37*). Amaranth flour has a high carbohydrate content (50%), especially starch (*2*). The hydrophilic nature of this polysaccharide negatively affects the permeability of the films (*40*). This is also suggested by the results found in this work. Glycerol is the most used plasticizer in film production, but its hydrophilic character favors the adsorption of water molecules and increases the space between the chains of the polymer. This leads to an increase of the WVP (*9*).

Regression analysis shows that the nanoparticles were also significantly affected by the WVP of the films. The permeability decreased when nanoparticle mass fraction increased (Fig. 3f). These results agree with prior works (*10*, *17*). Depending on their nature, nanoparticles can be impervious to mass transport, and thus pass through the film. The gases then must surround them to increase the diffusion time. Nanoparticles introduced into the films increase the tortuosity of the diffusion pathway (*41*). De Moura *et al*. (*8*) reported that nanoparticles can form hydrogen bonds between chitosan and the polymeric matrix to thus occupy the empty spaces of the porous matrix. This led to a decrease in the diffusion of water through the film.

The films had a moisture mass fraction of 20−30%. The resulting values are higher than those described by Tapia-Blácido *et al.* (*5*) and Villamán Diéguez *et al.* (*18*) for amaranth flour films without nanoparticles and with added nanoclay particles. The amaranth flour, glycerol and nanoparticles were found to have a significant effect on the moisture mass fraction of the films (p<0.05). It increased as the content of amaranth flour and glycerol increased (Fig. 3g). As previously mentioned, the hydrophilic nature of glycerol and the starch in amaranth flour can facilitate the formation of bonds with free OH groups. Similar results were reported by Thakur *et al*. (*37*). The moisture mass fraction increased with the mass fraction of glycerol and nanoparticles (Fig. 3i). Fundo *et al*. (*42*) concluded that high contents of chitosan/glycerol increased moisture in the films due to higher viscosity. This generates greater retention of water molecules during the film drying. A seemingly opposite effect was observed where high moisture values were seen at low amounts of both variables (Fig. 3h).

The films had a water solubility of 53-63%. It reflects the water-resistance and integrity of the films, and thus lower values are required (*37*). The water solubility of the films was similar to those published by Tapia-Blácido *et al.* (*5*) but higher than that reported by Villamán Diéguez *et al*. (*18*) for amaranth flour films with nanoclay. Thus, the content of amaranth flour, glycerol and nanoparticles was statistically significant. The water solubility of the films decreased with increasing content of amaranth flour, glycerol and nanoparticles (Figs. 2j-l). These results suggest that the components have stronger interactions at higher amount, and that the water molecules cannot easily break these interactions. The results are similar to those reported by De Moura *et al*. (*43*).

#### Mechanical properties

**Fig. 4:**
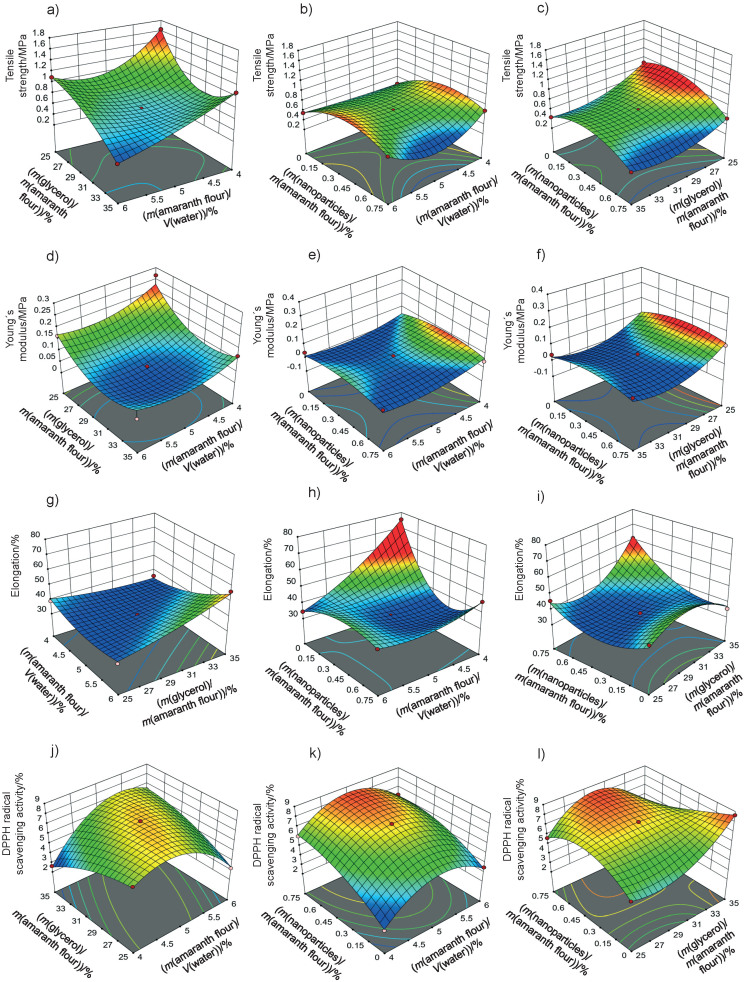
Response surface plots of the combined effects of independent variables on the: a-c) tensile strength, d-f) Young´s modulus, g-i) elongation and k-l) DDPH radical scavenging activity of amaranth flour films with chitosan particles loaded with chia extract

Mechanical properties are important to determine the resistance of the films. Fig. 4 shows the graphs of the response surface of tensile strength, Young’s modulus and elongation of the prepared films. The tensile strength values of the films were 0.33−1.63 MPa. These values are lower than reported for cellulose films (62.6 MPa) (*8*), fish gelatin films (11.8 MPa) (*10*), and zein films reinforced with chitosan particles (2.15 MPa) (*44*). They are better than the tensile strenght obtained for gelatin films treated with chitosan particles (0.16 MPa) (*14*). The tensile strength values are also lower than those reported by Tapia-Blácido *et al.* (*5*) (1.9−4 MPa) and Chandla *et al.* (*6*) (2.3−2.61 MPa) in amaranth flour films without nanoparticles and similar to those described by Villamán Diéguez *et al.* (*18*) (1.6 MPa) in amaranth flour films with incorporated clay particles.

This response was significantly influenced by glycerol and nanoparticle mass fraction (p<0.05). The tensile strength of the film was lower with increasing mass fraction of the glycerol (Figs. 4a and 4c). It interacts with the components of the polymeric matrix due to its hydrophilic nature, thus forming hydrogen bonds that generate greater plasticity in the films. Nanoparticles improved the tensile strength at mass fractions ranging from 0.15−0.6%. However, mass fractions higher than 0.6% generated a decrease in tensile strength (Fig. 4b). Nanoparticles can improve the tensile strength by creating new covalent and hydrogen bonds with the molecules of the polymeric matrix. This in turn increases the rigidity of the films (*45*). The homogeneous distribution, miscibility and compatibility of the nanoparticles with the polymeric matrix reinforce the film (*36*). In this study, the particles improve the tensile strength of amaranth flour films but only at certainmass fractions. This behavior was also described in tara gum and starch films with added chitosan particles (*9*, *17*). The addition of higher amount of nanoparticles in the matrix led to an agglomeration, and thus to the decrease of tensile strength. López-Rubio *et al*. (*46*) found that nanoreinforcement depends on the amount of particles. The increase in the content of the particles causes them to self-aggregate. As a result, there was a high viscosity, producing a lower tensile strength (*47*).

The Young’s modulus and elongation values of the films were 0.03−0.26 MPa and 28.89−61.11% respectively. The values obtained in the Young’s modulus response were lower than those reported for amaranth flour films without nanoparticles (90−233 MPa), those treated with clay nanoparticles (1.4−176.5 MPa) (*5*, *18*) and for gelatin films reinforced with chitosan nanoparticles (287−467 MPa) (*10*). The Young’s modulus was significantly affected by glycerol mass fraction (Table 3 and Figs. 4d-f). Films with a high tensile strength also had a higher Young’s modulus. De Moura *et al.* (*43*) used cellulose films that incorporate chitosan particles and found similar results. The high elastic modulus is indicative of a better film resistance.

The content of amaranth flour, glycerol and nanoparticles had a significant effect on the elongation of the films (p<0.05). The elongation of the films was greater than that reported for amaranth films without nanoparticles (10−47.3%), amaranth flour films with added clay particles (14−16.7%) (*5*, *18*), tara gum (35−40%) and zein (40%) films with added chitosan particles (*9*, *44*). However, it was lower than that reported in gelatin films with chitosan nanoparticles (152.63%) (*14*).

The films showed higher elongation with higher glycerol mass fractions (Fig. 4g). These results are consistent with the behavior observed for the tensile strength: films with greater resistance showed lower elongation. The added nanoparticles also modified the elongation of the films. The highest elongation was found with the highest values of both independent variables (glycerol and nanoparticles) (Fig. 4i). In contrast, the lowest elongation was seen in films with low values of amaranth flour and without nanoparticles (Fig. 4h). The particles decreased the elongation of the films but increased their resistance. Our results agree with Antoniou *et al*. (*9*), Hosseini *et al*. (*10*) and Vahedikia *et al*. (*44*).

#### Antioxidant properties

The antioxidant activities of the amaranth flour films treated with chitosan particles loaded with chia extract were 2.24−5.65% (Table 1). Statistically, this response was affected by the content of amaranth flour and particles (Table 3). The highest antioxidant activity was found in the films made with approx. 5% amaranth flour, which decreased with increasing content (Figs. 4j and 4k). Amaranth flour contains minerals, vitamins, proteins and phenolic-type compounds such as protocatechuic, *p*-hydroxybenzoic and caffeic acids with antioxidant activity (*48*). The nanoparticles also contributed to the antioxidant activity of the films. The highest values were found at nanoparticle mass fractions of 0.35−0.75% (Figs. 4k and 4l). The values of DPPH radical scavenging activity of the films were lower than those reported for gelatin films treated with chitosan particles loaded with green tea polyphenols (*49*), for gelatin films with added chitosan nanoparticles loaded with tea polyphenols generated by electrospray (*35*), and for nanocomposite films based on chitosan and gelatin loaded with chitosan nanoparticles (*50*). The differences in the antioxidant activity can be attributed to the content of nanoparticles added to the films, the concentration of the extract in the particles, encapsulated extract characteristics, and the type of the obtained nanoparticles (nanocapsules or nanospheres). Particles added to the films can release encapsulated bioactive compounds during storage, thus increasing the antioxidant activity of the films. Bao *et al*. (*49*) showed that the antioxidant activity of the films increased after 30 days of storage, and thus more studies should be carried out on the release of phenolic compounds from the nanoparticles added to the amaranth films.

#### Optimization of film formulation

Three of the main responses were chosen to optimize the formulation (content of amaranth flour, glycerol and nanoparticles) of the films: WVP, tensile strength and DPPH radical scavenging activity. These are the most important parameters for the functionality of the films. The films were defined to have minimum WVP values and maximum values of tensile strength and antioxidant activity. With these characteristics, they are recommended for the preservation of food. The optimal values were obtained from the independent variables with the Design Expert software v. 13 (*29*) and they were 4% amaranth flour, 25% of glycerol and 0.36% nanoparticles. The composite desirability value found was 0.85. The amounts obtained in the optimization were the lowest (lower limit) for the amaranth flour and glycerol. The values of the responses WVP, tensile strength and antioxidant activity predicted by the program for the films with the optimized formulation were 0.91·10^-7^ (g·mm)/(Pa·s·m^2^), 1.62 MPa and 6.43% respectively. These results are satisfactory in terms of costs because lower amounts of the polymer, plasticizer and nanoparticles could be used.

## CONCLUSIONS

In this research, response surface methodology was used to analyze the physical, barrier, mechanical and antioxidant activity properties of amaranth flour films treated with chitosan particles loaded with antioxidant chia extract. The considered independent variables (the content of amaranth flour, glycerol and nanoparticles) had different effects on the properties of the films. Glycerol content negatively affected the properties due to its hydrophilic nature and plasticizing capacity. The nanoparticles improved the tensile strenght, water vapor permeability and antioxidant capacity of the films compared to the films without nanosystems. Based on the model, the optimal formulation for preparation of the amaranth flour films with chitosan nanoparticles was 4% amaranth flour, 25% glycerol and 0.36% chitosan nanoparticles. This led to satisfactory water vapor permeability, tensile strength and antioxidant capacity. The results can be useful for the design of food packaging made of amaranth flour.
